# Aspects of yaw control design of an aircraft with distributed electric propulsion

**DOI:** 10.1007/s13272-022-00595-1

**Published:** 2022-07-14

**Authors:** Dennis Vechtel, Jan-Philipp Buch

**Affiliations:** German Aerospace Center (DLR), Institute of Flight Systems, Lilienthalplatz 7, 38108 Brunswick, Germany

**Keywords:** Hybrid-electric propulsion, Distributed propulsion, Yaw control, Flight simulator

## Abstract

Distributed electric propulsion (DEP) offers new options in aircraft design. Besides the optimization of the wing, another area of optimization is the vertical tail plane (VTP) and yaw control. The large number of engines significantly relaxes the one-engine-inoperative (OEI) case during take-off, which is mostly the sizing case for the VTP. This offers the possibility to reduce the VTP and rudder size to a certain amount. Also, the dynamics of electric motors offer the possibility to use differential thrust for yaw control. This can compensate at least some of the reduced rudder effectiveness coming from the smaller VTP size. In the framework of the German nationally funded project SynergIE, different aircraft designs of a hybrid-electric regional aircraft were investigated. Three aircraft concepts with 2, 6 and 12 propellers were designed in the project, for which reasonable minimum VTP sizes were investigated. For the 12-propeller aircraft, the investigations showed that the VTP could be reduced by 50%, still allowing the compensation of OEI during take-off and the generation of sideslip angle during crosswind operations. This reduction in VTP size results in a reduction of the block fuel by about 4%. For the 12-engine aircraft, a 6-degrees-of-freedom simulation model was developed including flight control laws for yaw control using the rudder and differential thrust. Virtual flight tests were performed in a full-flight simulator. The tests generally showed a good agreement with the theoretical results from the handling quality analysis but also outlined deficiencies in aircraft handling at low speed with full flaps. The use of a flight simulator at this early stage of aircraft design has proven to be a useful tool to investigate such unconventional designs.

## Introduction

Distributed propulsion offers a variety of areas for optimization of the aircraft configuration. The most prominent area of optimization is the wing design. Due to the large portion of the wing that lies in the slipstream of the propellers, the propellers generate a significant amount of additional lift. For this reason, the wing chord can be reduced, which reduces the wing area and increases the aspect ratio. Both save aerodynamic drag and structural weight. For a typical propeller-driven regional aircraft, the possible savings of required energy from the application of distributed electric propulsion with reduced wing chord lie in the range of about 10–20% compared to a electrically driven two-propeller aircraft of same size [1]. A prominent example for such a wing and propulsion design is the NASA experimental aircraft X-57 [2]. Besides the wing design, other areas might be subject to optimisation as well, enabled by the use of distributed propulsion.

Within the German nationally funded project SynergIE, the benefits of distributed propulsion for a hybrid-electric regional aircraft were investigated. Here, aircraft with 2, 6 and 12 electrically driven propellers were investigated. In the project, a major part was, of course, the design and optimization of the propulsion system [3], but also the influence of distributed propulsion on the overall aircraft design and many other aerodynamic or flight mechanical aspects were investigated.

Aircraft with electric propulsion, no matter whether they are designed with a fully electric propulsion system (e.g., battery as energy source) or like in this project a hybrid-electric propulsion system (with fuel as energy source and a gas turbine as generator for electric power), suffers the same problem, namely a heavier propulsion system compared to conventional propulsion. For this reason, such aircraft needs to be subject to an even much more in-depth optimization to outweigh the disadvantages in mass.

The main focus of this paper lies on the optimization of the aircraft’s yaw control. The sizing cases for the vertical tail plane (VTP) and the rudder are typically the critical engine failure during take-off, the generation of a sideslip angle suitable for crosswind landings and the general provision of directional stability.

For conventional aircraft, the critical engine failure during take-off is usually a case with one (critical) engine inoperative (OEI). The critical engine is typically the most outboard engine as it generates the largest yawing moment to be counteracted. It is obvious that with distributed propulsion, hence a larger number of engines, the critical engine failure during take-off is more relaxed, at least if still a failure of only one engine is considered to be the critical case. This needs to be assured by the propulsion system architecture design. Within the project, it was decided that still a single engine failure is to be considered the critical, hence sizing case.

By relaxing the OEI case, other cases, such as the directional stability and the associated dynamics of the Dutch roll, a typical Eigenmotion of aircraft, or the generation of sufficient sideslip angle for crosswind landings might become more crucial for the sizing of the VTP and rudder. However, the relaxation of the OEI case opens space for a possible reduction of the VTP size, saving both weight and aerodynamic drag.

On the other hand, the use of differential thrust for aircraft control might outweigh the reduced authority of the smaller rudder if the VTP is reduced in size.

To investigate the possibilities to optimize the VTP size and the aircraft’s yaw control enabled by the use of distributed propulsion, first a simplified flight mechanical study was performed. This study aims at assessing the potential to reduce the VTP size and to use differential thrust for aircraft control. The results of this study were fed back into the overall aircraft design process. Finally, a more sophisticated six-degrees-of-freedom simulation model was developed for the hybrid-electric 12-propeller aircraft, which was implemented in a full-flight simulator to investigate the flying qualities of the aircraft with pilots in the loop.

Using piloted flight simulator investigations within the aircraft, pre-design phase is uncommon today. For conventional aircraft designs, such an effort might indeed be overdone. However, for the design of unconventional aircraft configurations, such as a design with hybrid-electric distributed propulsion, it might be useful to utilize a flight simulator already at such early design phases to identify unforeseen aircraft behavior that needs to be considered in the further aircraft design. This could prevent a surprising emerge of adverse aircraft behavior during later design stages, when the necessary changes in the aircraft design are much more costly.

The work presented here contributes to enabling electrically powered aircraft by increasing the overall efficiency of the aircraft concept. Besides the design of an unconventional yaw control, the presented work outlines shortcomings of the investigated aircraft configuration in terms of handling qualities. By addressing them, future work may be able to overcome these shortcomings.

This paper describes at first the simplified flight mechanical study to assess the potential to reduce the VTP size and to use differential thrust for flight control, followed by the description of the six-degrees-of-freedom simulation model developed within the project. Finally, the virtual flight test in a full-flight simulator is described, showing a good example of the usefulness of such investigation within the pre-design phase.

## Simplified flight mechanical study

For the assessment of the optimization potential of yaw control including the VTP size and the use of differential thrust for yaw control, various aircraft configurations were investigated. The reference aircraft was the so-called TPR70, a conventional 70-seater regional aircraft designed by DLR within a previous CleanSky2 project [4]. In addition to the TPR70, hybrid-electric configurations with 2, 6 and 12 propellers were investigated. For the VTP layout, a T-tail and a conventional tail were examined for all hybrid-electric variants. For the 6-propeller and 12-propeller configurations, variants with a smaller conventional VTP were investigated.

**Fig. 1:**
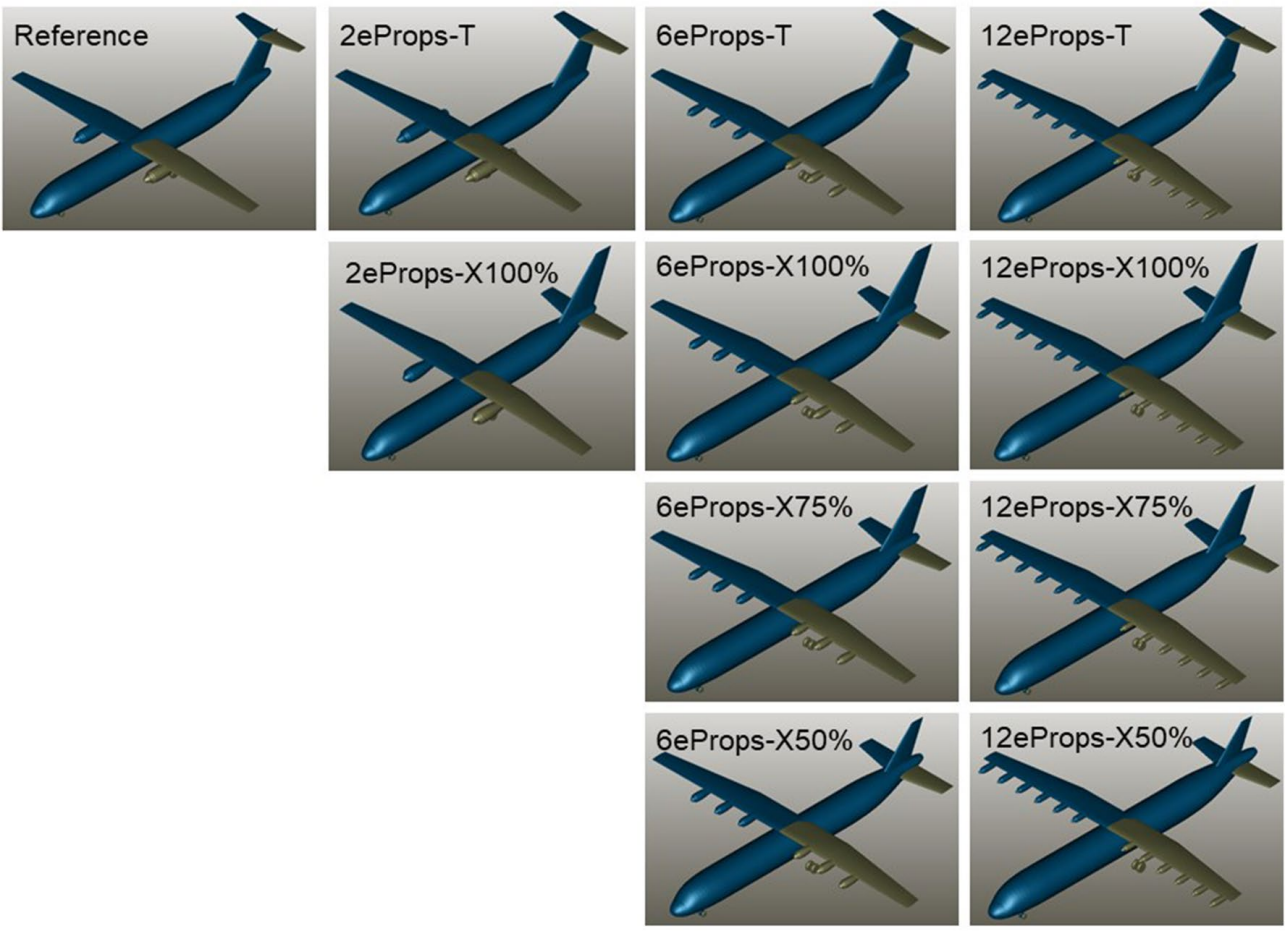
Analyzed aircraft configurations

Figure 1 shows 3D views of the different aircraft configurations investigated in the simplified study. As can be seen in Fig. 1, the conventional tails were investigated with 100%, 75% and 50% of VTP area compared to the reference aircraft.

The reasons for choosing a conventional tail for the variation of the VTP size instead of a T-tail were the bigger geometrical constraints for the reduction of the VTP size of a T-tail. To allow a better aerodynamic comparison, the variation of the VTP size should be performed with the same aspect ratio. As with a T-Tail, the minimum tip chord is given inherently by the chord length of the horizontal stabilizer, the reduction of the VTP size with the same aspect ratio is rather limited. For this reason, a conventional tail was chosen here, accepting the typical drawbacks of such a tail design for high-wing configurations.

It should be noted that for the different aircraft configurations investigated here, the variation of the VTP size does not only affect the drag and mass of the VTP but that it also has an effect on the whole aircraft design by considering all snowball effects.

### Derivative model

During aircraft pre-design, typically no detailed aerodynamic data are available that are required for flight mechanical investigations. For this reason, a simplified derivative model was set up here. The calculation of damping and control derivatives was based on handbook methods using the limited geometrical information available.

For the calculation of some derivatives, the lift curve slope of the wing, the HTP and the VTP are required. These are solely a function of the respective aspect ratio [5]:

1 $${C}_{L\alpha }=\frac{2\pi\Lambda }{2+\sqrt{4+{\Lambda }^{2}}}.$$

Please note that for the VTP the ${C}_{L\alpha }$ is denoted with ${C}_{Y\beta }$. However, physically, it is the same.

Roll damping is one of the most important parameters for the investigation. The following equation is used, using only the aspect ratio of the wing *Λ* [6]:

2 $${C}_{lp}=-\frac{1}{4}\cdot \frac{\pi\Lambda }{\sqrt{\frac{{\Lambda }^{2}}{4}+4}+2}.$$

The coupling of yaw and roll motion is mainly caused by the rudder. To estimate this derivative, a strip method for the VTP is used. This means that the VTP is divided into different strips for which the local sideforce is evaluated. The side forces of each strip are used to calculate the rolling moment generated by the VTP due to a yaw rate

3 $${C}_{lr}=\frac{\partial {C}_{l,\mathrm{VTP}}}{\partial r}.$$

The rolling moment of the VTP is the sum of the rolling moments of all *i* strips

4 $${C}_{l,\mathrm{VTP}}=\sum_{i}\left(-{C}_{Y,i}\cdot \frac{{z}_{i}}{B/2}\right).$$

The side force of each VTP strip is calculated by

5 $${C}_{Y,i}=-{C}_{Y\beta ,\mathrm{VTP}}\cdot {\beta }_{i}(r)\cdot \frac{{S}_{\mathrm{strip}}}{S}\cdot \mathrm{ellip}\left({z}_{i}\right)\cdot \mathrm{cos}{\varphi }_{\mathrm{VTP}},$$

assuming an elliptical lift distribution.

The factor for the elliptic lift distribution is calculated by

6 $$\mathrm{ellip}= \frac{4}{\pi }\mathrm{sin}\left({\mathrm{cos}}^{-1}\left(\frac{{z}_{i}}{{H}_{\mathrm{VTP}}}\right)\right).$$

The rolling moment due to a sideslip angle is divided into two parts. According to [7], the portion coming from the wing is given as a fixed value for an unswept wing, while the portion from the VTP is calculated again using a strip approach following Eqs. (4) and (5).

7 $${C}_{l\beta }={\Delta C}_{l\beta ,\mathrm{wing}}+\frac{\partial \left({C}_{l,\mathrm{VTP}}\right)}{\partial \beta }.$$

According to [7], the individual part from the wing is set to

8 $${\Delta C}_{l\beta ,\mathrm{wing}}= -0.075.$$

The yawing moment due to roll rate is estimated according to [7] by

9 $${C}_{np}=-\frac{{C}_{L}}{4}\left(1-\frac{3}{2+\sqrt{{\Lambda }^{2}/4+4}}\right).$$

The yaw damping comes mainly from the vertical stabilizer and is estimated using a strip method again

10 $${C}_{nr}=\frac{\partial {C}_{n,\mathrm{VTP}}}{\partial r}.$$

The yawing moment of the VTP is the sum of the yawing moment of all *i* strips

11 $${C}_{n,\mathrm{VTP}}=\sum_{i}\left({C}_{Y,i}\cdot \frac{{x}_{i}}{B/2}\right).$$

The lateral forces on the VTP are calculated using Eq. (5).

The yawing moment due to a sideslip angle, the so-called directional stability, comes mainly from the vertical stabilizer. Here, again, a strip model is used in analogy to yaw damping.

The lateral force due to sideslip is also estimated using the strip method. The calculation is analogous to that of the directional stability. Since the fuselage also has an influence on the lateral force, this portion is estimated in the same way, with the only difference that a different lateral force gradient is used for the fuselage strips. Here, a fixed value based on previous experience is used

12 $${C}_{Y\beta ,\mathrm{fuselage}}= 0.8.$$

The rolling moment of the rudder is calculated according to [8] from the given geometries and lever arms together with the lateral force gradient according to Eq. (1).

13 $${C}_{l\zeta }={\mathrm{0,8}\cdot C}_{Y\beta ,\mathrm{VTP}}\cdot \tau \cdot \mathrm{cos}\left({\varphi }_{\mathrm{VTP}}\right)\cdot \frac{{S}_{\mathrm{VTP}}}{S}\cdot \frac{{l}_{f}}{B/2}.$$

The effectiveness factor *τ* is also calculated according to [8] by

14 $$\tau =1-\frac{{\Theta }_{f}-\mathrm{sin}\left({\Theta }_{f}\right)}{\pi },$$

15 $${\Theta }_{f}={\mathrm{cos}}^{-1}\left(2\cdot {c}_{f}/c\right)-1.$$

The relative chord length of the rudder *c*_*f*_/*c* is assumed here to be 32%.

According to [6], the following applies to the yawing moment of the rudder:

16 $${C}_{n\zeta }=-{\frac{\partial \beta }{\partial \zeta }\cdot C}_{Y\beta ,\mathrm{VTP}}\cdot \mathrm{cos}\left({\varphi }_{\mathrm{VTP}}\right)\cdot \frac{{S}_{\mathrm{VTP}}}{S}\cdot \frac{{x}_{\mathrm{VTP}}}{B/2},$$

with

17 $$\frac{\partial \beta }{\partial \zeta }=1-\frac{2}{\pi }{\mathrm{cos}}^{-1}\left(\sqrt{\frac{{c}_{f}}{c}}-\sqrt{\left(\frac{{c}_{f}}{c}\right)\cdot \left(1-\frac{{c}_{f}}{c}\right)}\right).$$

The rolling moment of the ailerons is calculated analogously to Eq. (13) by

18 $${C}_{l\xi }=-2\cdot \left({\mathrm{0,8}\cdot C}_{L\alpha ,\mathrm{wing}}\cdot \tau \cdot \frac{{y}_{ob}-{y}_{ib}}{B/2}\cdot \mathrm{ellip}\left({l}_{f}\right)\cdot \mathrm{cos}\left(\varphi \right)\cdot \frac{{l}_{f}}{B/2}\right),$$

with the effectiveness factor *τ* according to Eqs. (14) and (15) and the assumption of an elliptical lift distribution. The factor for elliptic lift distribution $ellip$ is calculated here according to Eq. (6) using the wing span $B$.

The results of the calculated derivates cannot be validated for the investigated aircraft configurations as no real aircraft exists against which the quantitative values could be validated. However, the equations were also applied to other aircraft where validated models were available; hence, accurate numbers for damping and control derivatives were available. Comparison to the A320 and the Do-328 showed deviations of up to 30%. However, for aircraft pre-design, such a magnitude of deviation must be expected given the low fidelity of the applied methods.

### Potential for reduction of VTP size

An important design criterion for the sizing of the VTP is the dynamics of the so-called Dutch roll, which is one of the Eigenmotions of an aircraft. It is mainly influenced by the directional stability (*C*_*nβ*_) and the yaw damping (*C*_*nr*_). For the dynamics of the Dutch roll, handling quality boundaries were defined with regard to damping and natural frequency [9]. These boundaries are defined in [9] for the different handling quality levels and are depicted in Table 1.

**Table 1:** Handling quality boundaries for the Dutch roll [9]

	min. *D*_DR_	min. *ω*_*0,*DR_
Level 1	0.08	0.4
Level 2	0.02	0.4
Level 3	0	0.4

The estimation of natural frequency and damping of the Dutch roll is performed by [10]

19 $${\omega }_{0,\mathrm{DR}}=\sqrt{\frac{\rho }{2}\cdot {V}^{2}\cdot \frac{S\cdot B/2}{{I}_{z}}\cdot {C}_{n\beta }},$$

20 $${D}_{\mathrm{DR}}=-\sqrt{\frac{\rho }{8{\cdot I}_{z}}\cdot S\cdot {\left(B/2\right)}^{3}\cdot {C}_{n\beta }}\cdot \frac{{C}_{nr}}{\sqrt{{C}_{n\beta }}}.$$

The quantitative results are shown in Table 2 for the investigated configurations. As can be seen in Table 2, the natural frequency of the Dutch roll is well above the required minimum value from Table 3 and is therefore sufficient. Indeed, the damping is not sufficient for both configurations with a 50% reduced VTP size. However, this applies only to aircraft with a direct flight control without any flight controller. With an electronic flight control system, the handling qualities can be adapted. In principle, the limit values given in [9] only apply to aircraft with conventional, direct flight controls. Aircraft with a controller-augmented, electronic flight control system must demonstrate sufficient handling qualities with a functioning flight control system. Therefore, a degradation of the handling qualities to level 2 in the event of a failure of the flight control system may be acceptable if the probability of such occurrence is low. For this reason, all configurations are considered acceptable from the handling quality point of view.

**Table 2:** Natural frequency and damping of the Dutch roll of the investigated configurations

VTP size	6eProps			12eProps		
	100%	75%	50%	100%	75%	50%
ω_0,DR_	0.76	0.66	0.54	0.77	0.67	0.48
*D*_DR_	0.088	0.076	0.063	0.090	0.078	0.055

**Table 3:** Data of the investigated aircraft configurations

	Reference	2eProps		6eProps				12eProps			
		T-Tail	X-Tail	T-Tail	X-Tail			T-Tail	X-Tail		
			100%		100%	75%	50%		100%	75%	50%
MTOM [kg]	21,598	27,070	27,070	26,054	26,054	26,054	26,054	26,118	26,118	26,118	26,118
*T*_TO_ [*N*]	55,700	67,900	67,900	45,600	45,600	45,600	45,600	42,100	42,100	42,100	42,100
*S* [m^2^]	62.208	69.117	69.03	66.519	66.955	66.422	65.916	66.689	67.703	66.98	54.977
*B* [m]	27.001	28.203	28.185	27.667	27.758	27.647	27.542	27.703	27.912	27.763	25.153
*Λ* [–]	11.72	11.51	11.51	11.51	11.51	11.51	11.51	11.51	11.51	11.51	11.51
*S*_VTP_ [m^2^]	10.998	12.672	15.39	12.437	15.319	11.526	7.7536	12.674	15.992	11.953	6.0903
*H*_VTP_ [m]	3.6016	3.7017	4.8064	3.7017	4.7952	4.1593	3.4115	3.7017	4.8995	4.2357	3.0235
*Λ*_VTP_ [–]	1.179	1.081	1.5	1.102	1.5	1.5	1.5	1.081	1.5	1.5	1.5
*x*_VTP_ [m]	12.96	12.96	12.96	12.96	12.96	12.96	12.96	12.96	12.96	12.96	12.96

In addition to the dynamics of the Dutch roll, an engine failure during take-off is also relevant for the design of the VTP. The rudder must at least be able to compensate the yawing moment caused by the engine failure. This means that the yawing moment from the maximum rudder deflections has to be larger than (or at least the same as) the yawing moment from the engine(s)

21 $${N}_{{\zeta }_{\mathrm{max}} }\begin{array}{c}!\\ \ge \\ \end{array}{ N}_{\mathrm{Eng}}.$$

The yawing moment from the thrust in the event of *n* engine failures is

22 $${N}_{\mathrm{Eng}}=\sum_{{n}_{\mathrm{Eng},\mathrm{inop}}}\left(\frac{T}{{n}_{\mathrm{Eng}}}\cdot {y}_{\mathrm{Eng}}\right).$$

For the overall thrust *T*, the take-off thrust at *V* = 0 m/s cannot be used here, since the critical phase for an engine failure during take-off is at higher speeds. Therefore, the take-off thrust is too large, but the go-around thrust at minimum speed is the more realistic value. For this reason, and in absence of any better thrust model, the go-around thrust is used here instead of the take-off thrust.

The following applies to the maximum yawing moment of the fully deflected rudder:

23 $${N}_{{\zeta }_{\mathrm{max}}}={C}_{n\zeta }{\cdot \zeta }_{\mathrm{max}}\cdot \frac{\rho }{2}\cdot {V}^{2}\cdot S\cdot \frac{B}{2}.$$

**Fig. 2:**
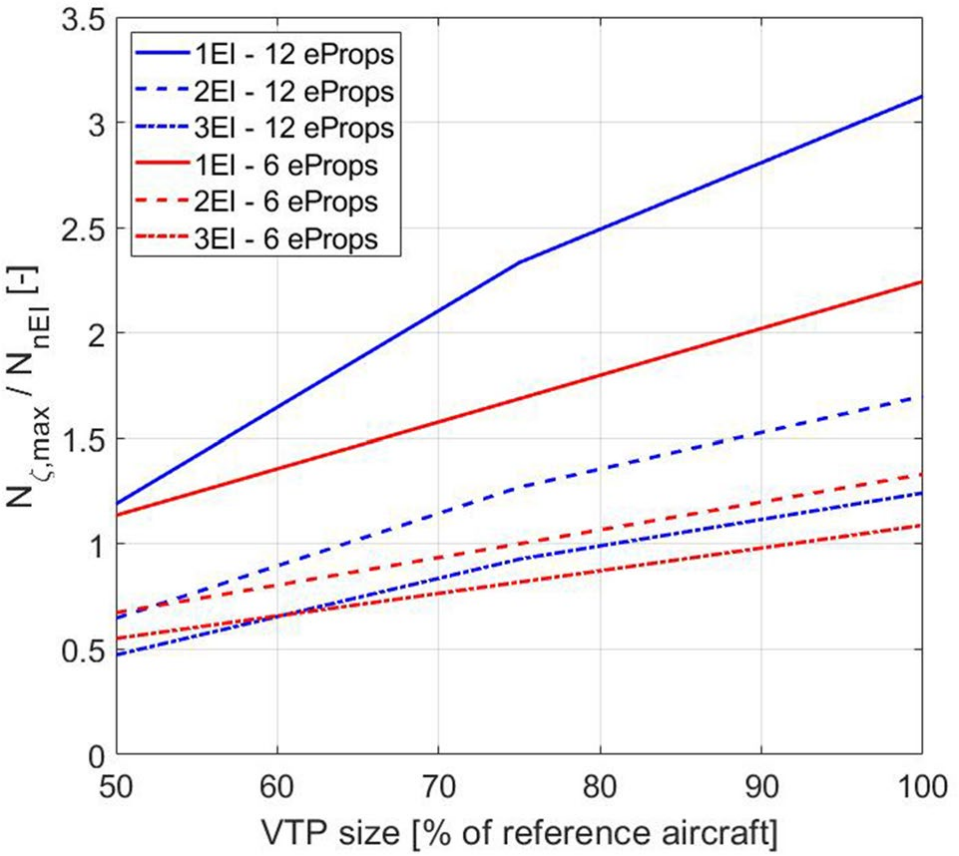
Capability of the 6- and 12-propeller configurations with different VTP sizes to compensate for 1,2 or 3 engine failures

Figure 2 shows the results for one, two and three engine failures (1,2,3EI—engine inoperative) for the investigated configurations with conventional tail and 12 and 6 propellers.

It can be seen that for the 12-propeller aircraft with a single engine failure, the VTP of 100% size in comparison to the reference aircraft is oversized by a factor of more than 3. This VTP and the associated rudder are able to compensate even 3 engine failures on the 12-propeller aircraft. For the 12-propeller aircraft, a reduction of the VTP size by 50% in comparison to the reference aircraft can still compensate one engine failure.

Within the project, it was decided that even for distributed electrical engines, only one engine failure should be considered as critical. For this reason, the 12-propeller configuration with the VTP reduced by 50% in comparison to the reference was chosen for a more detailed investigation as described in Sect. 3.

Compared to the 12-propeller aircraft with the 100% VTP, the reduction by one-half of the VTP area results in a 4% lower required block fuel for the 1,000 NM design mission.

### Potential to use differential thrust for flight control

The existence of twelve propeller engines generally allows for using differential thrust for flight control. To which extent differential thrust can actually be used for flight control and can compensate the smaller effectiveness of the rudder is investigated here based on simple models.

The propellers on both wings of the investigated aircraft configurations are counter-rotating, minimizing torque effects in case of symmetric thrust settings. For differential thrust settings, the torque effects of the small propellers of the 12-propeller configuration can be expected to be minor compared to the aerodynamic effects from the slipstream. For this reason, torque effects are disregarded here, concentrating only on aerodynamic effects of the slipstream.

The local increase in lift and drag in the slipstream of each propeller is calculated by means of Eqs. (24) and (25) as outlined in Sect. 3.

It must be understood that the yawing moment from differential thrust comes mainly from the thrust differences and that the change of drag in the slipstream of each propeller only has a minor effect on the yawing moment. In addition to that, differential thrust settings also generate a rolling moment. This rolling moment is generated by the local change of lift in the slipstream of the propeller. For this reason, each propeller generates a different amount of yawing and rolling moment for a given thrust.

**Fig. 3:**
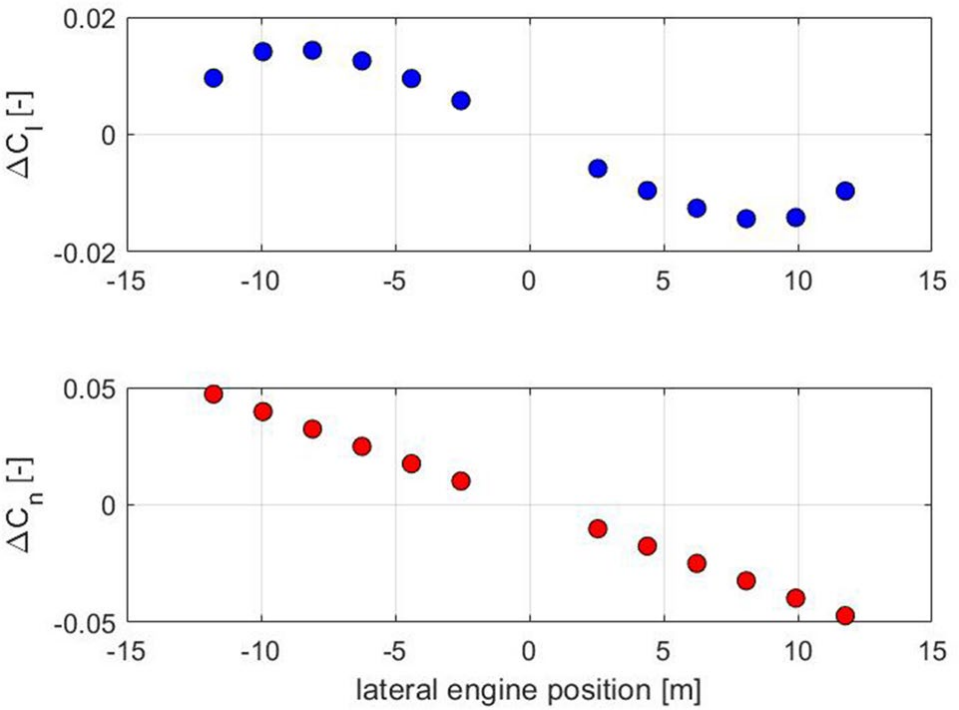
Rolling and yawing moment coefficients of the individual engines at take-off thrust (12eProps)

Figure 3 shows the rolling and yawing moment coefficients of all engines of the 12-propeller configuration at take-off thrust. It can be clearly seen that the absolute values of the yawing moments are significantly larger than those of the rolling moments. It can also be seen that the yawing moment increases nearly linearly with the lateral lever arm, while the rolling moment is a function of the (elliptic) lift distribution and thus decreases again toward the wing tip and has its maximum at approx. 2/3 of the wingspan.

**Fig. 4:**
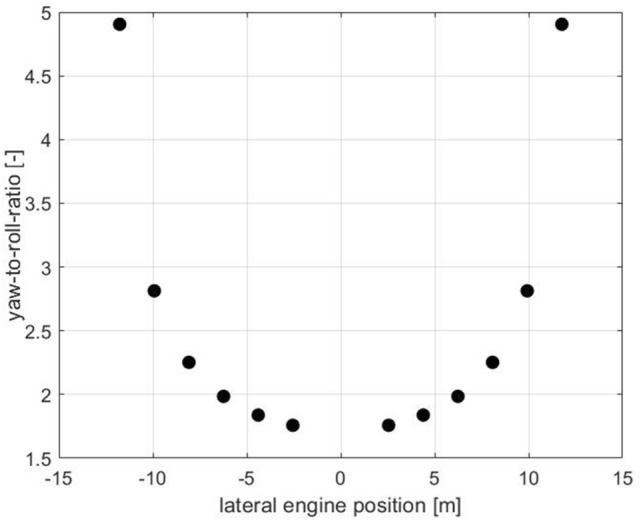
The yaw-to-roll ratio of each engine (12eProps)

Figure 4 shows the yaw-to-roll ratio of each engine for the 12-propeller configuration. Here, it can be seen again that in every case, the engines generate more yawing moment than rolling moment (ratio > 1). Due to the different curves of rolling and yawing moment, the ratio of yawing to rolling moment increases significantly toward the wing tip.

From Fig. 4, it can be seen that to generate a rolling moment, it is better to use the inboard engines to generate as little undesired yawing moment as possible. On the other hand, the outer engines should be used to generate a yawing moment with as little rolling moment as possible. Generally, various thrust settings are possible that generate different rolling moments at the same yawing moment and vice versa.

**Fig. 5:**
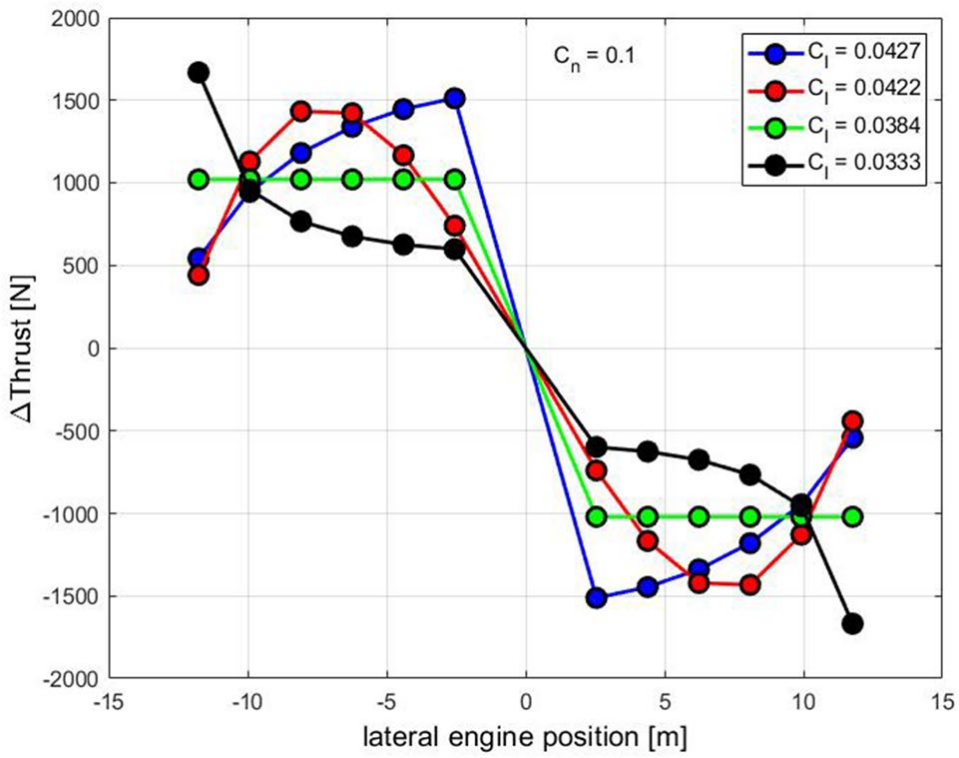
Rolling moment coefficients for different differential thrust settings and constant yawing moment coefficient (12eProps)

Figure 5 shows some exemplary thrust settings and the resulting rolling moment coefficients for the 12-propeller configuration. Here, the same yawing moment coefficient of *C*_*n*_ = 0.1 is generated in all cases.

Figure 5 shows that the blue thrust setting provides the largest rolling moment, but also requires a significantly larger additional thrust than, for example, the green thrust setting, which applies a constant additional thrust for all engines. By redistributing the thrust even more inwards, thrust distributions can be found that generate even larger rolling moments, but at the price of even higher additional thrust. On the other hand, the black thrust setting with the largest additional thrust on the outer engines provides the lowest rolling moment and should therefore be used for yaw control.

## Simulation model development

**Fig. 6:**
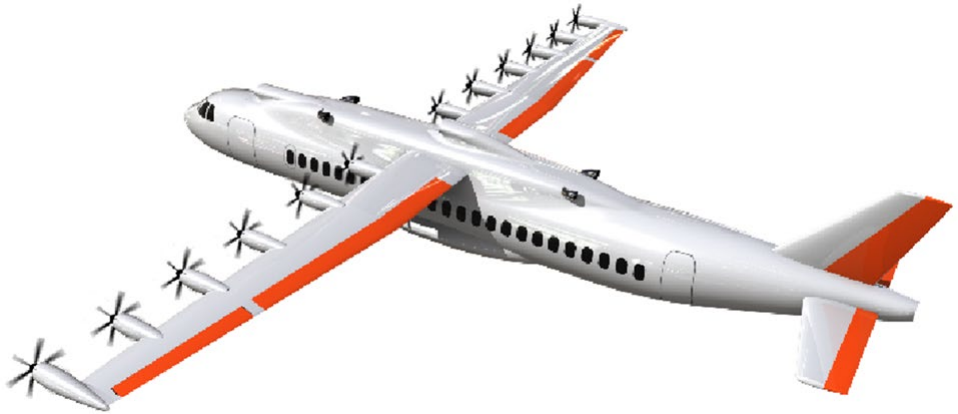
3D-sketch of the investigated hybrid-electric 12-propeller configuration

For the 12-propeller configuration with the 50% smaller VTP, a six-degrees-of-freedom model is developed for further investigation in a full-flight simulator. Figure 6 shows a 3D-sketch of this aircraft.

In the SynergIE project, the aircraft pre-design data exchange language Common Parametric Aircraft Configuration Schema (CPACS) is used, which provides data in an xml format. For using CPACS data to set up an aircraft simulation, a dedicated CPACS-based simulation software tool for six-degrees-of-freedom aircraft simulations was developed by the DLR Institute of Flight Systems called CPACS-Oriented Aircraft Simulation Tool (COAST). COAST is implemented in Matlab/Simulink^®^ and uses specific wrapper functions to load the aircraft data from the xml file into the simulation framework. In principle, the modeling in COAST largely follows the model approach described in [11]. Wherever CPACS deviates from this, the modeling of COAST follows the CPACS standard.

For the simulation model developed in SynergIE, project-specific changes had to be made to the basic COAST model. These changes mainly concern the aerodynamics model, the engine model and the landing gear model.

As during the aircraft pre-design phase, no detailed aerodynamic model is available, the tools LiftingLine and HandbookAero provided by the DLR Institute of Aerodynamics and Flow Technology via the Remote Component Environment (RCE) workflow environment were used to generate the aero maps as defined in the CPACS standard. These aero maps comprise the basic aerodynamics of the aircraft as a function of the angle of attack, the sideslip angle, Reynolds and Mach number, the roll, yaw and pitch rate and the control surface deflections.

For an aircraft like the 12-propeller configuration, in which a large part of the wing area lies in the wake of the propeller, the thrust-dependent influence on the aerodynamics must be modeled. The thrust-dependent increment of lift and drag is calculated for each of the i engines by

24 $$\mathrm{d}{c}_{fx,i}=\frac{{S}_{\mathrm{strip}}}{S}\cdot \left(\frac{{T}_{i}}{\frac{\rho }{2}{V}^{2}{S}_{\mathrm{Prop}}}{\cdot c}_{fx}\right),$$

25 $$\mathrm{d}{c}_{fz,i}=\frac{{S}_{\mathrm{strip}}}{S}\cdot \left(\frac{{T}_{i}}{\frac{\rho }{2}{V}^{2}{S}_{\mathrm{Prop}}}\cdot {c}_{fz}\cdot \frac{4}{\pi }\cdot \mathrm{sin}\left({\mathrm{cos}}^{-1}\left(\frac{{2y}_{i}}{B}\right)\right)\right).$$

For the calculation of the incremental lift coefficient components, an elliptical lift distribution is assumed, with which the total lift coefficient of the basic aerodynamics is weighted at the respective span position.

The total amount is summed up for all engines. The incremental parts of the coefficients of rolling, pitching and yawing moments are calculated from the force coefficients with the respective lever arms.

To be able to simulate landings, the simulation model requires a model of the aerodynamics of the high-lift system. The high-lift system is kept relatively simple and is designed as a plain flap with a maximum deflection angle of 20°. Since a large portion of the high-lift system lies in the slipstream of the propellers, it is assumed that a plain flap is sufficient. The high-lift system was not designed using CFD, but was only calculated in 2D. Based on the 2D calculations, equations for the angle-of-attack-dependent, local lift, drag and pitching moment coefficients were determined.

The high-lift aerodynamics model is designed as a strip model, since parts of the high-lift system are subjected to the additional flow velocity of the propeller slipstream and some parts (between the propellers) are not. The additional flow velocity in the wake of the propellers is calculated with

26 $$d{V}_{i}=\sqrt{\frac{{T}_{i}}{\frac{\rho }{2}{S}_{\mathrm{Prop}}}+{{V}_{i}}^{2}}-{V}_{i}.$$

The additional velocity from the yaw rate r is also considered for the calculation of the inertial velocity *V*_*i*_ of each strip. For the calculation of the effective angle of attack, the roll rate *p* is also considered.

The engine model used here differs from the original engine model of COAST. Coefficients for thrust and torque were stored in lookup tables as a function of the blade pitch angle *β* and the advance rate *J*. Thrust *T* and Torque *Q* are calculated by

27 $$T={c}_{T}\left(J,\beta \right)\cdot {n}^{2}\cdot {{D}_{\mathrm{Prop}}}^{4}\cdot \rho ,$$

28 $$Q={\frac{1}{2\pi }\cdot c}_{P}\left(J,\beta \right)\cdot {n}^{2}\cdot {{D}_{\mathrm{Prop}}}^{5}\cdot \rho .$$

The blade tip Mach number Matip is calculated by

29 $${Ma}_{\mathrm{tip}}=\frac{a}{\sqrt{{V}^{2}+{\left({D}_{\mathrm{prop}}\cdot n\cdot \pi \right)}^{2}}}.$$

The circumferential speed *ω* acts as a control variable for the engine model.

30 $$\omega =\int \frac{1}{{I}_{xx}}\left({Q}_{\mathrm{CMD}}-Q\right)\mathrm{d}t.$$

The revolutions *n* are calculated from this by

31 $$n=\frac{\omega }{2\pi }.$$

A propeller speed governor controls the pitch angle of the propeller blades *β* depending on the blade tip Mach number. The governor is designed as a PD controller with limits between 20° and 65° blade pitch angle.

To be able to simulate landings, the COAST model had to be expanded by a landing gear model, which is not included in the baseline COAST model. The landing gear is modeled as a three-point undercarriage, in which all three gear struts are modeled as an oleo-pneumatic spring-damper combination. The main landing gear also comprises brakes and the nose landing gear can be steered. The entire landing gear model corresponds to the modeling according to [12, 13]. The adjustment of the spring-damper characteristics was done qualitatively on the basis of the MTOM.

For the development of the flight controller, the flying characteristics of the uncontrolled aircraft were analyzed for two flight points: a flight point for cruise flight (Ma 0.5, altitude: 8000 m, clean configuration) and a flight point for landing (Ma 0.16, altitude: 304 m, landing configuration). The base aircraft generally has a poorly damped short period and phugoid mode for the flight points. The lateral motion shows an unstable but slow, and therefore easily manageable, spiral motion. The damping of the Dutch roll fulfills the requirement of damping according to [9] for both trim points.

The basic controller architecture was designed in analogy to the Airbus controller architecture. The controller for the longitudinal motion is designed as a modified C* control law. For the sake of simplicity, a thrust controller has been omitted. To improve the handling qualities as well as due to the reduced size of the VTP, a sideslip controller was designed for the lateral motion. This is intended to improve the handling qualities, which are impaired by the reduced rudder size, using differential thrust, especially in crosswind landings during de-crab. A roll rate controller was designed for the rolling motion.

The equation of the implemented C*-law is

32 $${\eta }_{\mathrm{cmd}}={C}_{\mathrm{cmd}}^{*} {h}_{\eta {C}^{*}}+{C}^{*}{k}_{\eta {C}^{*}}+\frac{1}{s}\left[{C}_{\mathrm{cmd}}^{*}-{C}^{*}\right]{j}_{\eta {C}^{*}}+{\dot{\Theta }}_{s}{k}_{\eta q},$$

with

33 $${C}^{*}=\Delta {n}_{z,\mathrm{cmd}}+ {\dot{\Theta }}_{s}\cdot \frac{{V}_{m}}{g},$$

and

34 $$\Delta {n}_{z,\mathrm{cmd}}=\left({n}_{z}-1\right)-\left(\frac{\mathrm{cos}\left(\Theta \right)}{\mathrm{cos}\left(\phi \right)}-1\right).$$

An auto-trim function ensures that the HTP is moved in such a way that the elevator is returned to its center position.

The normal law consists of a roll rate controller and a slip angle controller. The associated control laws for aileron and rudder deflection are

35 $${\xi }_{\mathrm{cmd}}={p}_{\mathrm{cmd}}{h}_{\xi p}+{p}_{kb}{k}_{\xi p}+\frac{1}{s}\left({p}_{\mathrm{cmd}}-{p}_{kb}\right) {j}_{\xi p},$$

36 $${\zeta }_{\mathrm{cmd}}={\beta }_{\mathrm{cmd}}{h}_{\zeta \beta }+\beta {k}_{\zeta \beta }+{1/s\left({\beta }_{\mathrm{cmd}}-\beta \right)+r}_{kb}{k}_{\zeta r}+{\xi }_{i,\mathrm{Err}}{k}_{\zeta {\xi }_{i,\mathrm{Err}}}.$$

When the pedals are deflected, a sideslip angle is commanded. The roll angle of the aircraft remains unchanged by the roll rate regulator if the pilot does not command a lateral deflection of the sidestick at the same time. This makes it possible to control the sideslip angle independently of the roll angle, which is seen as advantageous, especially when correcting the windward angle (de-crab) during landing.

The controller was designed qualitatively and exclusively for the two flight points described above (cruise with flaps retracted and landing with flaps fully extended). The controller gains are scheduled with the true airspeed.

In addition to the so-called normal law, with active basic controller, a possibility was created to be able to fly the aircraft without the basic controller, the so-called direct law. In the direct law a direct, linear coupling between sidestick and elevator/aileron was implemented, as would also be the case with a conventional aircraft. The pedals were also connected directly to the rudder.

The rudder command of the sideslip angle controller is used both on the rudder and to generate differential thrust across the twelve distributed engines. The thrust distribution in spanwise direction is implemented linearly, increasing outwards. The reason for this is to shift the ratio of the generated yawing moment to the generated rolling moment in favor of yaw, as described in Sect. 2.3.

It should be clear, that the developed simulation model of the 12-propeller aircraft configuration cannot be validated by any means. This should always be considered when interpreting the results from the simulations.

## Virtual flight tests

Unfortunately, due to the restrictions following the COVID-19 pandemic, the virtual testing of the 12-propeller configuration in the full-flight simulator could not be carried out as planned. The COVID-19-related closure and limited usability of the flight simulator only allowed for one test with a DLR test pilot.

### The AVES-simulator

**Fig. 7:**
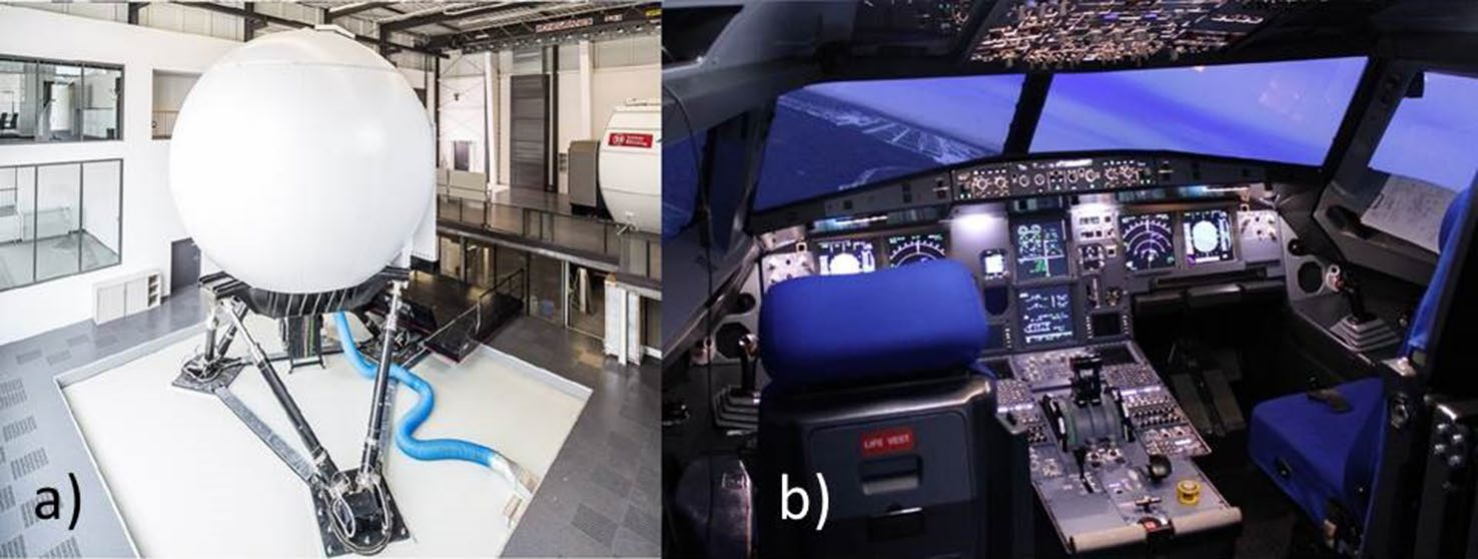
The AVES motion simulator with the A320 cockpit **a** simulator cabin on the hexapod motion system, **b** cockpit view in the simulator cabin

The AVES (Air Vehicle Simulator) simulator center is operated by the DLR Institute of Flight Systems since 2013 [14]. The simulator center consists of a fixed-base and a motion-based simulator with exchangeable cockpits of the A320 and the EC135. Figure 7a shows the motion-based simulator of the AVES from the outside. The main part is the large dome, which enables an enormous visual projection area of 240° in horizontal and 95° in vertical direction. The large vertical extent of the visual projection area is a requirement of the helicopter simulation, since helicopters require a much larger external view than fixed-wing aircraft. Another detail in Fig. 7 is the motion system, which consists of an electro-pneumatic hexapod system. Figure 7b shows the interior view of the AVES A320 cockpit. The cockpit is a replica of the A320 cockpit and represents the original in almost every detail. In the rear part of the cockpit (not visible in Fig. 7), an operator station for simulation control is implemented.

The entire AVES software was developed by the DLR Institute of Flight Systems (with the exception of the control of the motion platform). The flight simulation with flight control system, autopilot, flight management system, flight warning computer, system simulation as well as the simulator control software and the software for the external visual display was developed by the DLR Institute of Flight Systems. This guarantees maximum flexibility for scientific purposes.

To be able to simulate an aircraft model in AVES, further simulation modules, such as the simulation of the cockpit hardware (switches, levers, sidesticks, thrust levers), the displays, the external view or the simulation control, are necessary. The provision of these simulation modules in the stage of aircraft pre-design is limited and most of the required data cannot be provided here.

As a consequence of the technical software simplifications, the A320 cockpit used here only provided a few generic functions. The sidesticks, the pedals, the thrust levers, the flap lever and a simplified version of the primary flight display and navigational display were functional for the virtual flight tests. All other cockpit controls were not functional. The selection of the mode of flight control laws (normal or direct law) was implemented via the operator station and was set by the operator manually.

Due to the COVID-19-related unavailability of the AVES, the simulator integration was carried out as far as possible via remote connections. Without this, the pilot test would not have been feasible under the given conditions. However, this did not allow the full scope of integration tests to be carried out, which resulted, for example, in a restricted configuration option for the high-lift system (restriction to Conf 0 and Conf Full instead of all flap configurations).

### The piloted trials

Due to the COVID-19-related restrictions and the resulting very short integration and testing time, the main focus of the virtual flight tests was the determination of the general flying qualities.

Different manoeuvers were defined, which were flown at different altitudes and different high-lift configurations. The flight characteristics were assessed using the Cooper–Harper Rating Scale [15]. For this, limits must be defined for a specific flight task, within which the flying qualities can be considered desired or adequate.

The following limits were defined for the maneuvers carried out here:

- altitude: adequate ± 100 ft; desired ± 50 ft.
- speed: adequate ± 10 kts; desired ± 5 kts.

No limits have been defined for maintaining the heading.

Generally, ratings from 1 to 3 are referred to as level 1 handling qualities. Ratings of 4–6 correspond to handling qualities of level 2 and ratings 7–8 to level 3. A rating of 10 means that the aircraft is at least partially uncontrollable under the relevant test conditions.

The following test points were defined:

- Normal Law: 8000 ft, 250 kts, clean config
- (TP2.1) speed change to 220 kts
- (TP2.2) 30° right turn with 25° bank angle
- (TP2.3) speed change to 250 kts
- (TP2.4) climb to 9000 ft with constant heading and speed
- (TP2.5) 30° left turn with 25° bank angle
- (TP2.6) descend to 8000 ft with constant heading and speed
- (TP2.7) steady heading sideslip
- Direct Law: 8000 ft, 250 kts, clean config
- (TP2.8) Speed change to 220 kts
- (TP2.9) 30° right turn with 25° bank angle
- (TP2.10) Climb to 9,000 ft with constant heading and speed
- Normal Law: 3000 ft, 100 kts, config full
- (TP3.1) speed change to 130 kts
- (TP3.2) 30° right turn with 25° bank angle
- (TP3.3) speed change to 110 kts
- (TP3.4) climb to 4000 ft with constant heading and speed
- (TP3.5) 30° left turn with 25° bank angle
- (TP3.6) descend to 3000 ft with constant heading and speed
- (TP3.7) steady heading sideslip
- Direct Law: 3000 ft, 100 kts, config full
- (TP3.8) Speed change to 130 kts
- (TP3.9) 30° right turn with 25° bank angle
- (TP3.10) Climb to 4000 ft with constant heading and speed

The flight points marked “Normal Law” were carried out with the basic flight controller engaged. The flight points marked “Direct Law” were carried out with the uncontrolled aircraft.

The flight points TP2.1 to TP2.7 with retracted landing flaps (clean configuration) and activated flight control (normal law) are widely rated with level 1 handling qualities. Only the turns were rated with slightly degraded handling qualities (level 2). On the one hand, this was due to a somewhat sluggish aircraft reaction in the roll axis and difficulties in maintaining the exact altitude while rolling. According to the pilot, this can certainly be compensated by more experience to the behavior of the aircraft and corresponding training. When interpreting the results, it must also be considered that this is a preliminary design level and that the accuracy of the simulation is accordingly low. It can be assumed that the flight points TP2.1 to TP2.7 are not a problem with regard to the handling qualities. The flight points TP2.8 to TP2.10 in clean configuration and with the flight controller disengaged (direct law) were consistently rated with level 2 handling qualities, partly close to the limit of level 3, as maintaining altitude and speed within the limits defined as adequate was only possible with a very high workload of the pilot. The Dutch roll, which is less damped due to the small VTP, was less of a problem, but the weakly damped short period resulted in high demands on the pilot's workload. One reason for this was certainly the linear characteristic between stick and elevator deflection that was implemented here. With a little less pitch authority around the zero position, the pilot would probably be able to control the pitch of the aircraft more precisely. Unfortunately, this could not be tested due to the COVID-19 situation and the associated restrictions in the use of the simulator. It cannot be decided at this point whether the degraded handling qualities in direct law would be acceptable in terms of certification in the unlikely event that the normal law fails. However, degraded handling qualities are permissible in today’s aircraft in the event of an improbable failure of the normal law.

The test points TP3.1 to TP3.7 with fully extended landing flaps and activated flight controller (normal law) showed the characteristic deficits of the configuration, which certainly need further revision. The strong coupling between thrust and lift makes it very difficult for the pilot to maintain altitude and speed. The pure horizontal flight was not considered a separate test point, but it turned out that it was difficult for the pilot to maintain altitude and speed adequately, since every change in thrust to maintain speed is accompanied by a change in the aircraft’s vertical speed. It must be emphasized here that the nz controller of the flight controller’s pitch normal law should counteract this, as it wants to maintain a vertical load factor of 1 without any pilot's stick input. However, due to the high dynamics of the engines, hence a fast change in the load factor when changing thrust, the nz controller is not capable of maintaining unaccelerated flight vertically. This fact must be considered when designing the nz control of the pitch normal law.

With the current controller behavior, the pilot must therefore always keep an eye on the pitch position and thrust (pitch and power). For this reason, the maneuvers associated with large changes in thrust (change in speed TP3.1 or climb TP3.4) were rated with level 3 handling qualities, as the pilot was not able to acquire altitude and speed within the limits defined as adequate. At this point, an example should be given to describe the very unintuitive aircraft behavior with full flaps. If a climb is initiated, the increase in thrust results in a noticeable increase in lift and, as a result, a vertical load factor that cannot be compensated by the flight controller. The resulting climb rate can thus be generated solely by increasing the thrust. Since a multiple of the aircraft’s weight is not required when climbing (lift ≈ weight), the aircraft must be pitched down to maintain the same lift at a lower angle of attack. Otherwise it is not possible to maintain the speed. This means that to initiate a climb, the pilot has to increase thrust and at the same time pitch down. With this radically changed pilot technique, the test pilot was able to adjust his control behavior to the characteristics of the aircraft to such an extent that he could evaluate the handling qualities with level 2 after some practice. With such practice, heading changes were no longer a problem for the pilot, as they could be flown without a change in thrust, and were therefore rated level 1. This is certainly also due to the fact that the pilot had adjusted to the aircraft’s behavior after the significantly more demanding other manoeuvers and so the turns appeared comparably easy to him.

However, the flight points TP3.8 to TP3.10 in full configuration and in direct law showed that the aircraft is partially uncontrollable in this configuration. Here, the effect of the reduced rudder size was evident. While the yaw controller of the normal law (also using differential thrust for yaw control) was able to compensate for the lower directional stability and the associated only weakly damped yawing motion, this posed a problem for the manual control. Since the pilot in direct law only has the reduced rudder available for the yaw control, the authority of the yaw control was insufficient to fly the aircraft with an acceptable level of pilot workload. The pilot was only able to control the aircraft in the yaw axis with very high workload. Due to this extreme workload, the pilot often lost other parameters out of sight, such as altitude or speed. For this reason, the flight behavior with full flaps and in direct law must be considered uncontrollable. The handling qualities in the yaw axis can certainly be regarded as level 3 and therefore only as degraded, but in combination with the demanding coupling of thrust and lift the entire task is to be regarded as uncontrollable. A yaw damper which is acting independently from the normal law and that is also active in direct law could provide a remedy here.

Landing was not rated using the Cooper–Harper rating scale but only qualitatively. In principle, it was possible to land the aircraft in normal law, even as the maximum possible sink rate was relatively limited due to the relatively high idle thrust (approx. 700 ft/min maximum). It must be emphasized that this high idle thrust was only due to the accuracy level of the preliminary design and the very rough engine model, which was limited in terms of blade pitch angle. Since the aircraft therefore predominantly flies the approach in idle, the problems mentioned above like the thrust-lift coupling are not very pronounced. It can be expected that the exact adherence to a glide path is more difficult with lower idle thrust and a resulting higher thrust level.

When interpreting the results, it must be considered that the design of the high-lift system was not the focus in the project and was only modeled from 2D polars. The maximum deflection of the flap of 20° was just estimated as a reasonable value. The simulator tests have shown that the thrust-lift coupling at this deflection angle is too large to enable acceptable flight behavior in all regimes. It is therefore urgently recommended to focus more on this aspect in further investigations of this (or similar) aircraft configurations. The high-lift system should also be designed using CFD to map the thrust-lift coupling more precisely. In addition, various intermediate deflection angles should be investigated.

Another means to cope with the strong coupling of thrust and lift could be the use of the trailing edge flap as a direct lift control flap. This means, that if the flap is actuated fast enough, it could compensate the change in lift from a change in thrust by changing its deflection angle, hence maintaining the current lift.

## Conclusion

Benefits from distributed propulsion on yaw control and the sizing of the VTP have been assessed for a hybrid-electric 70-seater regional aircraft with 12 electrically driven propeller engines. A flight mechanical pre-design study using a simplified derivative model showed a potential to reduce the VTP size by 50% due to the distributed propulsion. Furthermore, the potential to use differential thrust for yaw control was assessed. With this VTP reduced by one-half, the required block fuel for the 1000 NM design mission could be reduced by 4%.

Based on the results of the simplified pre-study, a six-degrees-of-freedom simulation model of the 12-propeller aircraft with the small VTP (area reduced to one-half) was developed and integrated into a full-flight simulator. The simulation model incorporated a basic flight controller with a C*-law in the pitch axis, a roll rate command controller for the roll axis and a sideslip angle controller for the yaw axis, which uses rudder and differential thrust for yaw control. A virtual flight test was performed with a test pilot in the AVES full-flight simulator of the DLR Institute of Flight Systems in Braunschweig to investigate the flying qualities of the aircraft.

The simulator trial revealed that the developed yaw control worked sufficiently to counteract the reduced effectiveness of the smaller VTP and rudder. In direct law, when no controller is active and differential thrust is not used for improving the yaw stability, the handling qualities were indeed degraded, but still acceptable, as typically the case that the aircraft is flown without any controller assistance is an unlikely failure case nowadays.

However, the strong coupling of thrust and lift turned out to be a more fundamental degradation of aircraft handling, especially in landing configuration with the flaps fully deployed. The pitch controller of the flight control system was not able to compensate for such strong changes in vertical load factor in case of a change in thrust. This required a totally changed and unintuitive flying technique of the pilot. Without the flight controller, in direct law, the aircraft was uncontrollable due to the coupling of thrust and lift in combination with the low stability in the yaw axis, which together leads to excessive plot workload.

Due to the early phase of aircraft development, the aircraft simulation models developed here could not be validated. For this reason, the results obtained should be interpreted with care. The accuracy of the model could not be quantified; however, due to the low fidelity of the used methods (especially to generate the aerodynamic tables), errors of 10–20% are likely to occur. Nevertheless, the basic effects of aircraft behavior can be expected to be represented properly by the simulation model.

The aim of the investigation was to assess potentials and to identify shortcomings of the aircraft configuration. This was perfectly shown by the discovery of the strongly degraded handling qualities due to the strong coupling of thrust and lift in landing configuration with full flaps. This effect was neither expected to be so pronounced, nor was it the focus of the investigation, which mainly dealt with the yaw control of the aircraft.

For a further development of this or similar aircraft, the strong coupling of thrust and lift should be considered from the very beginning of the design. The high-lift system requires a detailed design to reduce the coupling with full flaps as much as possible. Furthermore, the basic flight controller needs to be adapted in a way that it can cope with the quick change of the vertical load factor in case of thrust changes. Maybe, a feed-forward control incorporating the (for this purpose fast actuated) trailing edge flaps as direct lift control flaps could be a solution to improve the aircraft handling.

Having said this, the use of piloted flight simulator trials within the pre-design phase of an unconventional aircraft design has proven to be a useful means to identify shortcomings of the aircraft design at a very early stage.

## Abbreviations

AVES: Air Vehicle Simulator
CFD: Computational Fluid Dynamics
COAST: CPACS-Oriented Aircraft Simulation Tool
CPACS: Common Parametric Aircraft Configuration Schema
DEP: Distributed electric propulsion
DLR: German Aerospace Center
HTP: Horizontal tail plane
MTOM: Maximum Take-Off Mass
NASA: National Aeronautics and Space Administration
OEI: One Engine Inoperative
RCE: Remote Component Environment
VTP: Vertical tail plane

## List of symbols

$a$: Speed of sound (m/s)
$$B$$: Wing span (m)
$$c$$: Wing chord (m)
$${c}_{f}$$: Flap chord (m)
${C}_{f,x}$: Longitudinal aerodynamic force coefficient (–)
${C}_{f,z}$: Vertical aerodynamic force coefficient (–)
${C}_{l}$: Rolling moment coefficient (–)
${C}_{l\beta }$: Sideslip-roll derivative (–)
$${C}_{lp}$$: Rolldamping derivative (–)
${C}_{lr}$: Yaw-roll derivative (–)
${C}_{l\zeta }$: Aileron roll derivative (–)
${C}_{l\xi }$: Rudder roll derivative (–)
$${C}_{L}$$: Lift coefficient (–)
${C}_{L\alpha }$: Lift curve gradient (–)
${C}_{n}$: Yawing moment coefficient (–)
${C}_{n\beta }$: Sideslip-yaw derivative (–)
${C}_{np}$: Roll-yaw derivative (–)
$${C}_{nr}$$: Yawdamping derivative (–)
${C}_{n\zeta }$: Rudder yaw derivative (–)
$${C}_{Y}$$: Sideforce coefficient (–)
${C}_{Y\beta }$: Sideslip-sideforce-derivative (–)
$$D$$: Damping (–)
$${D}_{\mathrm{prop}}$$: Propeller diameter (m)
$\mathrm{ellip}$: Factor for elliptic lift distribution (–)
$$H$$: Height (m)
${l}_{f}$: Spanwise flap length (m)
${I}_{z}$: Yaw inertia (kg/m^2^)
$$J$$: Advance ratio (–)
$$n$$: Rotational speed (1/s)
${n}_{\mathrm{eng}}$: Number of engines (–)
${n}_{z}$: Vertical load factor (–)
$$Ma$$: Mach number (–)
$N$: Yawing moment (Nm)
$$p$$: Roll rate (°/s)
$$Q$$: Torque (Nm)
$$r$$: Yaw rate (°/s)
$$S$$: Area (m^2^)
$$T$$: Thrust (N)
$${T}_{\mathrm{TO}}$$: Takeoff thrust (N)
$$V$$: True airspeed (m/s)
$$x$$: Longitudinal position (m)
${x}_{\mathrm{VTP}}$: Lever arm of VTP (m)
$$y$$: Lateral position (m)
$$z$$: Vertical position (m)
$$\beta$$: Sideslip angle (°)
$\eta$: Elevator deflection angle (°)
$\zeta$: Aileron deflection angle (°)
$$\Theta$$: Pitch angle (°)
$$\Lambda$$: Aspect ratio (–)
$\xi$: Rudder deflection angle (°)
$\rho$: Air density (kg/m^3^)
$$\mathrm{\varphi }$$: Sweep angle (°)
$$\Phi$$: Bank angle (°)
$\tau$: Flap effectiveness factor (–)
$$\omega$$: Angular velocity (1/s)
$${\omega }_{0}$$: Natural frequency (1/s)
